# Adipo-Epithelial Transdifferentiation in In Vitro Models of the Mammary Gland

**DOI:** 10.3390/cells13110943

**Published:** 2024-05-30

**Authors:** Jessica Perugini, Arianna Smorlesi, Samantha Acciarini, Eleonora Mondini, Georgia Colleluori, Chiara Pirazzini, Katarzyna Malgorzata Kwiatkowska, Paolo Garagnani, Claudio Franceschi, Maria Cristina Zingaretti, Christian Dani, Antonio Giordano, Saverio Cinti

**Affiliations:** 1 Department of Experimental and Clinical Medicine, Center of Obesity, Marche Polytechnic University—United Hospitals, 60126 Ancona, Italy; j.perugini@univpm.it (J.P.); asmorlesi@gmail.com (A.S.); samanthaacciarini@gmail.com (S.A.); ele_mondini@yahoo.it (E.M.); g.colleluori@univpm.it (G.C.); maria.cristinazingaretti@alice.it (M.C.Z.); a.giordano@univpm.it (A.G.); 2 Department of Medical and Surgical Sciences (DIMEC), University of Bologna, 40126 Bologna, Italy; chiara.pirazzini5@unibo.it (C.P.); katarzyn.kwiatkowsk2@unibo.it (K.M.K.); paolo.garagnani2@unibo.it (P.G.); claudio.franceschi@unibo.it (C.F.); 3 IRCCS, Azienda Ospedaliero-Universitaria di Bologna, 40138 Bologna, Italy; 4 Laboratory of Systems Medicine of Healthy Aging, Institute of Biology and Biomedicine and Institute of Information Technology, Mathematics and Mechanics, Department of Applied Mathematics, N. I. Lobachevsky State University, 603005 Nizhny Novgorod, Russia; 5 Faculté de Médecine, CNRS, INSERM, iBV, Université Côte d’Azur, CEDEX 2, F-06107 Nice, France; christian.dani@univ-cotedazur.fr

**Keywords:** adipocytes, mammary gland, pregnancy, cellular transdifferentiation, cell culture

## Abstract

Subcutaneous adipocytes are crucial for mammary gland epithelial development during pregnancy. Our and others’ previous data have suggested that adipo-epithelial transdifferentiation could play a key role in the mammary gland alveolar development. In this study, we tested whether adipo-epithelial transdifferentiation occurs in vitro. Data show that, under appropriate co-culture conditions with mammary epithelial organoids (MEOs), mature adipocytes lose their phenotype and acquire an epithelial one. Interestingly, even in the absence of MEOs, extracellular matrix and diffusible growth factors are able to promote adipo-epithelial transdifferentiation. Gene and protein expression studies indicate that transdifferentiating adipocytes exhibit some characteristics of milk-secreting alveolar glands, including significantly higher expression of milk proteins such as whey acidic protein and β-casein. Similar data were also obtained in cultured human multipotent adipose-derived stem cell adipocytes. A miRNA sequencing experiment on the supernatant highlighted mir200c, which has a well-established role in the mesenchymal–epithelial transition, as a potential player in this phenomenon. Collectively, our data show that adipo-epithelial transdifferentiation can be reproduced in in vitro models where this phenomenon can be investigated at the molecular level.

## 1. Introduction

Adipocytes are cells containing abundant cytoplasmic lipids. White adipocytes store fatty acids in the form of triglycerides that are released to accomplish the energy needs of the body during intervals between meals. Brown adipocytes burn fatty acids to produce heat to assure the body’s thermal homeostasis. Both functions are essential for individual survival. During the last decades, we have provided data supporting that in mice, as well as in humans, white and brown adipocytes are organized in tissues that form a distinct organ called the adipose organ [1]. In mammalian organs, different tissues interact and cooperate for a specific finalistic role. Our and others’ data strongly suggest that in the adipose organ such cooperation is related to the ability of the adipose tissues to convert into each other under specific physiologic conditions: during chronic cold exposure white adipose tissue (WAT) converts into brown adipose tissue (BAT) to increase thermogenesis, whereas during chronic positive energy balance, BAT converts into WAT to increase energy storage abilities to face unpredictable fasting periods [2]. Importantly, this functional cooperation partly arises from a new cell property: transdifferentiation [1,2]. Such phenomenon was recently proved by lineage tracing experiments [3]. Considering the specific role of BAT in energy expenditure, transdifferentiation holds significant therapeutic potential [4,5].

In females, a specific part of the thoracic subcutaneous fat in the case of humans, or the whole subcutaneous fat depot in the case of mice, has another important functional role: lactation. This specific fat depot looks morphologically very similar to that of males, with the only difference consisting of the presence of branched ducts infiltrating the whole breast and ending in nipples in females [6]. In women, the subcutaneous thoracic fat forming the two breasts is dominated by two nipples, whereas in mice five bilateral nipples dominate the anterior area of the whole subcutaneous fat. In adult virgin female mice, the volume of the breasts is composed of about 90% subcutaneous fat.

Alveologenesis, that is the development of milk-producing and milk-secreting glands, occurs only during pregnancy and lactation. Concomitantly to the increase in the number of mammary alveoli, there is a decrease in the adipocyte content, which is almost absent at the apex of lactation [6]. At the end of lactation, adipocytes progressively reappear in mice subcutaneous depot. At post-lactation day 10, almost all alveoli disappear, and the morphological appearance of the subcutaneous depot reverts to the virgin-like anatomy, where few and poorly branched ducts are located among numerous and well-differentiated adipocytes. Similar aspects are also evident in humans [7]. This highly plastic tissue rearrangement is currently thought to be the result of a lipid depletion in adipocytes during pregnancy which is followed by lipid re-filling in the post-lactation period. Adipocyte lipid depletion would allow alveoli to complete the milk production. It is believed that lipid-depleted adipocytes hide among the glands during lactation and that they are progressively refilled with lipids during the weaning period. Furthermore, apoptosis of alveolar epithelial cells in the post-lactation period would explain their disappearance [6].

In striking contrast, our electron microscope studies showed that lipid-depleted adipocytes are still visible even when all lipids are lost. Furthermore, this technique allowed us to document intermediate aspects of trandifferentiation of adipocytes into alveolar cells during pregnancy and vice versa in the post-lactation period [8]. Bromodeoxyuridine incorporation, lineage tracing, and explant experiments supported the hypothesis that a physiologically reversible adipo-epithelial transdifferentiation occurs in the breast during pregnancy, lactation, and the post-lactation period [8]. One of the most impressive aspects of adipo-epithelial conversion is that most newly formed alveolar cells (around day 18 of pregnancy) show a distinctive histological appearance, mainly characterized by the presence of a large amount of lipids in their cytoplasm. Therefore, they fulfill the general definition of adipocytes, and for this reason, we called them pink adipocytes [8]. Recently, a paper arguing against adipo-epithelial transdifferentiation in the mammary gland was published [9], but only lineage tracing was employed, and, in our experience, different models of transgenic mice can give different results, at least in different areas of the mammary glands.

The molecular mechanisms underlying reversible adipo-epithelial transdifferentiation are poorly known, but our published data pointed to osteopontin (SPP1), a paracrine factor secreted by ductal epithelial cells, playing a key role in this phenomenon [8].

In this study, we assessed the possibility of transdifferentiating mature adipocytes from mouse subcutaneous fat in vitro under experimental conditions mimicking pregnancy. Collectively, experimental conditions mimicking pregnancy in vitro induced loss of the adipocyte phenotype parallel to the acquisition of an epithelial one, with a molecular signature specific to milk-secreting mammary alveolar cells. Lastly, an analysis conducted on the supernatant from the co-culture system revealed mir200c as a possible mediator of the adipo-epithelial transdifferentiation, consistent with studies showing its ability to promote the mesenchymal–epithelial transition in different experimental settings [10,11].

## 2. Materials and Methods

### 2.1. Materials

Corning^®^ Matrigel^®^ Basement Membrane Matrix, progesterone, insulin, hydrocortisone, 3,39,59-triiodo-L-thyronine (T3), transferrin, ascorbic acid, biotin, and 2,2,2-tribromoethanol were purchased from Sigma-Aldrich (St. Louis, MO, USA). Type III collagenase was purchased from Worthington Biochemical (Freehold, NJ, USA) and type I collagenase was purchased from Invitrogen (Thermo Fisher Scientific, Waltham, MA, USA). Gentamicin, Dulbecco’s modified eagle’s medium (DMEM), Ham’s nutrient mixture F12, and fetal bovine serum (FBS) were obtained from Gibco (Thermo Fisher Scientific, Waltham, MA, USA). Human recombinant epidermal growth factor (EGF), human recombinant fibroblast growth factor 2 (FGF-2), and prolactin were obtained from Peprotech (Thermo Fisher Scientific, Waltham, MA, USA). Clear transwell inserts were purchased from Costar Corporation (Cambridge, MA, USA). Sterile tissue-culture plastic plates and culture flasks were purchased from Becton–Dickinson Labware (Franklin Lakes, NJ, USA).

### 2.2. Animals and Tissue Processing

Adult CD1 virgin female mice were housed in plastic cages under constant environmental conditions (12 h light/dark cycle, at 22 °C) with free access to standard chow diet and water. Handling was limited to cage cleaning. Their care was conducted according to Council Directive 2010/63/EU. All experiments were approved by the Italian Health Ministry (Authorisation no. 405/2018-PR). At 14 and 20 weeks of age, mice were sacrificed to isolate the mammary epithelial organoids (MEOs) (see below) and, respectively, mammary gland mature adipocytes. Specifically, they were anaesthetized with an overdose of 2,2,2-tribromoethanol, sacrificed by cervical dislocation and the left and right mammary glands were removed.

### 2.3. Isolation of Mammary Epithelial Organoids

Inguinal mammary glands were excised from CD1 female mice and epithelial organoids were isolated after mechanical and enzymatic disaggregation using Collagenase type III (2 mg/mL) as previously described [12]. Briefly, digested mammary glands were separated into fractions by centrifugation to exclude floating adipocytes and then filtered using nitex filters sized 530 mm and 60 mm in turn to remove large tissue fragments and single cells, respectively. MEOs that remained on the 60 mm filter were recovered and cultured on plastic plates for 4 h at 37 °C to allow the attachment and subsequent removal of residual stromal cells. As previously described, the number of cells in the organoid suspension was calculated using triplicate nuclei counts [13]. Once isolated, MEOs were embedded in Matrix Matrigel (80% in serum free medium), a solid basement membrane preparation which acts as a scaffold, rich in extracellular matrix proteins, including type IV collagen, laminin, proteoglycans, and growth factors, including fibroblast growth factor (FGF), epidermal growth factor (EGF), platelet-derived growth factor (PDGF), transforming growth factorβ (TGFβ), and insulin growth factor (IGF). Matrigel has been shown to provide a supportive microenvironment for various cell types, facilitating cell adhesion, migration, and differentiation. By incorporating Matrigel into our in vitro system, along with its diffusible growth factors, we aim to reproduce the in vivo environment, where the extracellular matrix undergoes constant remodeling and growth factors play a crucial role in differentiation processes [14].

### 2.4. Isolation of Mammary Mature Adipocytes

Unilocular mature adipocytes were obtained from inguinal mammary fat pads from CD1 female mice according to the technique used in Bjorntorp et al. [15]. Briefly, inguinal mammary glands were excised, minced, and digested at 37 °C for 1 h in HBSS with 2 mg/mL type I collagenase. A pure fraction of isolated floating adipocytes and a pellet containing the stroma-vascular fraction (SVF) were obtained by centrifugation at 150 g for 7 min. For cell culture, aliquots of fully differentiated adipocytes were suspended in DMEM/F12 and immediately used.

### 2.5. Transwell Model for Co-Culturing Mammary Epithelial Organoids and Adipocytes

A transwell co-culture model was set up based on the method described by Darcy KM. et al. [16,17], with some modifications, to study the reciprocal interactions between mammary adipocytes and MEOs under conditions mimicking the microenvironment of the in vivo mammary gland (Figure S1).

MEOs were cultured at 37 °C within a layer of Matrigel, in a 6-well plate, in serum- free DMEM/F12 and in the presence/absence of pregnancy hormones and growth factors to stimulate the extensive and sustained mitogenic, morphogenic, and lactogenic responses [18]. Hormones and growth factors were insulin (10 µg/mL), apotrasferrin (5 µg/mL), progesterone (1 µg/mL), hydrocortisone (1 µg/mL), prolactin (1 µg/mL), ascorbic acid (0.88 µg/mL), EGF (10 ng/mL), and gentamicin (50 µg/mL). Whereas growth factors are thought to allow cellular proliferation and differentiation [19], the hormones support the development of mammary organoids [17].

The day that the organoids were set up in Matrigel was considered day T0. The MEOs were let to grow and differentiate for 7 and 14 days. Transwell inserts were prepared at day 7 (T7) and 14 (T14) of the MEO cultures. One milliliter aliquots of mammary mature adipocytes were plated in each transwell insert, which was subsequently placed into the six-well plate containing MEOs embedded in Matrigel and co-cultured for 3 days in serum-free DMEM/F12 in the presence/absence of the pregnancy hormones (Figure S2). The inserts had a porous polyethylene tetraphthalate membrane that permits free flow of solutes—but not cells—between the two compartments. The membrane has a pore size of 0.4 mm and a very low protein binding affinity. The MEO controls were cultivated in wells with Matrigel alone to account for the possible release of diffusible substances (such as growth factors and cytokines) from the Matrigel. Finally, according to the experimental design (Figure S2), six experimental conditions were analyzed, as shown in Table 1.

### 2.6. hMADS Cell Culture and Treatments

Human multipotent adipose-derived stem cells (hMADS) were cultured as described previously [20,21]. In brief, hMADS grown in low-glucose (1 g/L) proliferation medium DMEM supplemented with 10% FBS and 2.5 ng/mL FGF-2 were used between the 16th and the 19th passage. To induce adipose differentiation, they were seeded in a proliferation medium on multi-well plates at a density of 4500 cells/cm^2^. When they reached confluence, FGF-2 was not replaced. The next day (day 0), cells were incubated in an adipogenic medium (serum-free proliferation medium/Ham’s F-12 medium) containing 10 µg/mL transferrin, 5 µg/mL insulin, 0.2 nM triiodothyronine, 100 µM 3-isobutyl-1-methylxanthine, 1 µM dexamethasone, and 100 nM rosiglitazone. Dexamethasone and 3-isobutyl-1-methylxanthine were not replaced from day 3 and rosiglitazone was not replaced from day 9. Cell lipid content was assessed at different time points by using Oil Red O staining. Treatments and biological assays were carried out on differentiated hMADS adipocytes from day 12 to day 15. Treatment duration and SPP1 and pregnancy hormone concentrations are reported in the Results section.

### 2.7. qRT-PCR

Total RNA was extracted with Trizol reagent (Thermo Fisher Scientific, Waltham, MA, USA), purified, digested with ribonuclease-free deoxyribonuclease, and concentrated using the RNeasy Micro kit (Qiagen, Milano, Italy) according to the manufacturer’s instructions. For determination of mRNA levels, 1 μg RNA was reverse-transcribed with the High-Capacity cDNA RT Kit with RNase Inhibitor (Thermo Fisher Scientific, Walthama, MA, USA) in a total volume of 20 μL. qRT-PCR was performed using TaqMan Gene Expression Assays and Master Mix TaqMan (Applied BioSystems, Foster, CA, USA). All probes (Table 2) were from Applied BioSystems. Reactions were carried out in a Step One Plus Real Time PCR system (Applied BioSystems) using 50 ng DNA in a final reaction volume of 10 μL. The thermal cycle protocol included initial incubation at 95 °C for 10 min followed by 40 cycles of 95 °C for 15 s and 60 °C for 20 s. For each sample, a control reaction was run without reverse transcriptase in the amplification mixture to rule out genomic contamination. Every sample was tested twice. In every experiment, negative controls were samples that did not contain the template. TATA box-binding protein (TBP) was used as an endogenous control to normalize gene expression. Relative mRNA expression was determined by the ΔCt method (2^−ΔΔCt^).

### 2.8. Western Blotting

Adipocyte lysates were obtained by using lysis buffer containing 50 mM Tris–HCl (pH 7.4), 1% NP-40, 1 mM EDTA, 150 mM NaCl, 1 mM sodium orthovanadate, 0.5% sodium deoxycholate, 0.1% SDS, 2 mM phenylmethylsulfonylfluoride and 50 mg/mL aprotinin. Protein concentrations were measured using the Bradford Protein Assay (Bio-Rad Laboratories, Segrate, Italy) after samples were centrifuged. Proteins were separated using SDS-PAGE and then moved using the Trans-Blot TurboTM Transfer system (Bio-Rad) to a nitrocellulose membrane. Membranes were visualized using Ponceau S solution (Santa Cruz Biotechnology, Dallas, TX, USA) in order to assess loading and transfer efficiency. Membranes were then blocked for 1 h at room temperature (RT) in TBS-Tween-20 (50 mM Tris-HCL [pH 7.6], 200 mM NaCl and 0.1% Tween-20) containing 5% non-fat dried milk and subsequently incubated overnight at 4 °C with the primary antibody (Table 3).

After washing in TBS-Tween-20 and incubation with the secondary antibody for 1 h at RT (Table 4), bands were visualized with the Chemidoc Imaging system using the ClarityTM Western ECL chemiluminescent substrate (all from Bio-Rad, Segrate, Italy). Quantitation of immunoreactive bands was performed using Image Lab 6.0.1 software (Bio-Rad, Segrate, Italy). Where appropriate, membranes were stripped, washed, and re-probed for total protein content.

### 2.9. miRNAseq Analysis in the Co-Culture Medium

According to the experimental design (Figure S1), miRNAseq analysis was performed in 18 samples obtained from 3 biological replicates of the six experimental conditions as mentioned in Table 1.

Small RNA was isolated from 250 µL of supernatant using the miRNeasy Serum/Plasma kit (Qiagen, Milano, Italy) that allows the isolation of cell-free total RNA molecules from approximately 18 nucleotides upwards. RNA was quantified by Qubit (Thermo Fisher Scientific, Waltham, MA, USA) and used for library preparation with NEXTflex Small RNA Seq Kit v3 (Bio Scientific, Sydney, Australia). An RNA fragment (21 nt), not matching any known sequence in miRbase, was included as a positive control. Briefly, total RNA was denatured, 4N Adenylated Adapters were added to the 3′ end, and the excess was removed. Then, NEXTflex™ 4N Adapters were added to 5′ end and the reverse transcription occurred, followed by PCR amplification. After a final purification, the libraries were quantified by Qubit and their quality was checked at the bioanalyzer (Agilent, High Sensitivity DNA Kit). All the samples were sequenced on Illumina HiSeq. The bioinformatic pipeline was carried out with R and Python. Reads from fastq files obtained from Illumina HiSeq were pre-filtered, and the 3′ adapter was trimmed with cutadapt. The remaining reads were aligned to the reference genome (Mus musculus primary assembly GRCm38 from Ensembl) with bowtie2 and annotated and counted with htseq-count provided within HTSeq Python package. Finally, differential analysis was performed with the R Bioconductor package edgeR. Three types of comparisons were performed: (1) transdifferentiating condition (A+MEO+H) vs. each single condition alone (A, A+H, A+MEO, A+Matrix, A+Matrix+H); (2) transdifferentiating condition (A+MEO+H) vs. all other joined conditions (A, A+H, A+MEO, A+Matrix, A+Matrix+H) and (3) transdifferentiating conditions (A+MEO+H and A+Matrix+H) vs. non-transdifferentiating ones (A, A+H, A+MEO, A+Matrix).

### 2.10. Electron Microscopy

After co-culture, MEOs were fixed in 2% glutaraldehyde plus 2% paraformaldehyde in 0.1 M phosphate buffer (pH 7.4) for at least 4 h, post-fixed in a solution of 1% osmium tetroxide and 1% potassium hexacyanoferrate (II), dehydrated in acetone and finally epoxy-resin embedded. Semi-thin sections (2 µm) were stained with toluidine blue. Thin sections obtained with an MT-X ultratome (RCM, Tucson, AZ, USA) were mounted on copper grids, stained with lead citrate, and examined with a CM10 transmission electron microscope (Philips, Eindhoven, Netherlands).

### 2.11. Statistical Analysis

All experiments were performed in triplicate. Results are reported as mean ± standard error of the mean (SEM). Comparisons between biological groups were performed with one-way analysis of variance (ANOVA). A *p*-value < 0.05 was considered as a statistically significant difference. All statistical analyses were performed with GraphPad Prism 8.4.2 software (GraphPad Software, Boston, MA, USA).

## 3. Results

### 3.1. Pregnancy Hormones Affect Molecular and Morphological MEO Differentiation

**Figure 1:**
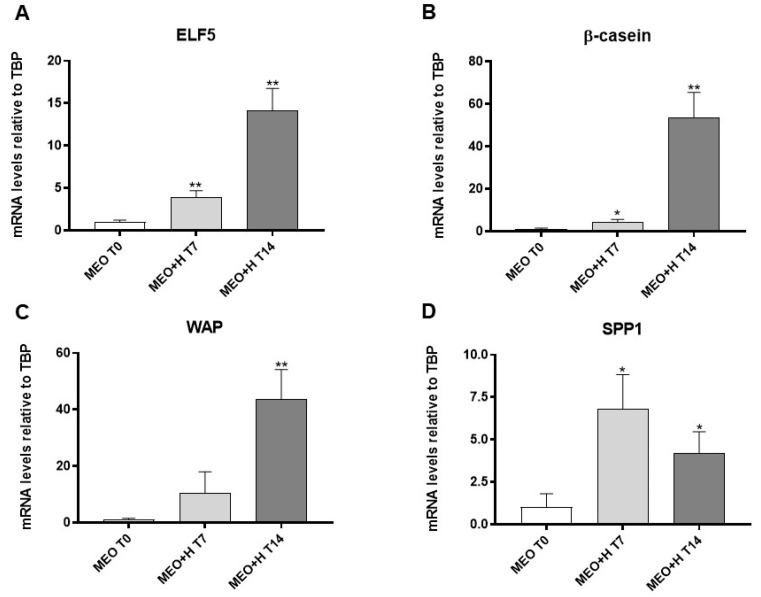
qRT-PCR analysis of ELF5 (**A**), β-casein (**B**), WAP (**C**) and SPP1 (**D**) expression in freshly isolated MEO (T0) and MEO at 7 and 14 days of culture under hormonal stimulation (H). Data (*n* = 3) are mean ± SEM; * *p* < 0.05, ** *p* < 0.01 compared with MEO T0. Data were analyzed using one-way ANOVA.

MEOs were grown for 7 and 14 days on Matrigel and exposed to pregnancy hormonal stimulation to develop alveolo-ductal 3D organoids. Functional differentiation was evaluated by qRT-PCR (Figure 1). Compared to non-treated MEOs (MEO T0), hormone-treated MEOs exhibited a significantly higher expression of ELF5 (Figure 1A) and β-casein (Figure 1B) after 7 days; the expression of these markers increased even more after 14 days, when a significant increase of whey acidic protein (WAP) was also detectable (Figure 1C). Hormone-treated MEOs showed a significantly higher expression of SPP1, a marker of pregnancy-dependent mammary gland ductal differentiation, after 7 and 14 days (Figure 1D). Notably, SPP1 is a secreted paracrine factor, which we found to be involved in alveolar development and adipo-epithelial transdifferentiation [8]. Wnt6 and RANK-L are also important paracrine factors for mammary alveologenesis and milk production [22,23]. Interestingly, hormone-treated MEOs also expressed significant levels of these genes (Figure S3).

**Figure 2:**
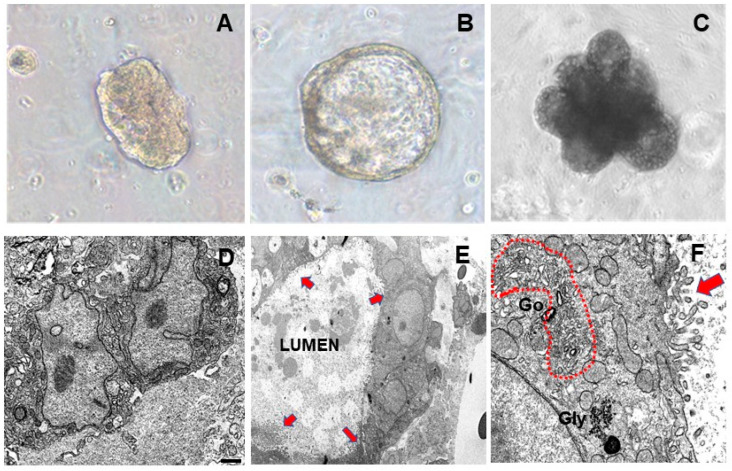
Phase contrast light microscopy of freshly isolated (T0) MEO embedded in Matrigel (**A**), MEO grown for 7 days (**B**) and 14 days (**C**) in the presence of pregnancy hormones in a serum-free culture medium. Electron Microscopy of MEO without hormones (**D**); MEO after pregnancy hormones addition (**E**): alveolar differentiation with lumen and microvilli (arrows); (**F**): enlargement of an alveolar structure showing hypertrophic Golgi complex (dotted area), glycogen cluster (Gly), and well-developed microvilli (arrow), all signs of alveolar differentiation. Bar: 1 µm in (**D**) 2 µm in (**E**) and 0.3 µm in (**F**).

Differentiation of hormone-treated MEOs toward alveolar structures was also assessed by phase contrast light microscopy. As shown in Figure 2, MEOs progressively enlarged and formed luminal cavities surrounded by polarized luminal epithelial cells under hormonal stimulation (7 days) (Figure 2B); after 14 days, MEOs gave rise to honeycomb-like structures formed by multiple alveolar cavities (Figure 2C), strongly resembling the 3D aspect of fully differentiated mammary gland alveoli. In our experimental setting, longer-term incubation (T21) of organoids was associated with degeneration aspects and apoptotic responses [24].

Electron microscopy images showed that non-treated MEOs were formed by epithelial cells without specific signs of differentiation (Figure 2D). In striking contrast, hormone treatment induced an alveolar-like differentiation in MEO cells which appeared joined together to form luminal cavities (Figure 2E) and exhibited distinctive ultrastructural aspects of alveolar differentiation including hypertrophic Golgi complex, glycogen clusters, and well-developed microvilli (Figure 2F).

Collectively, these data show that MEOs cultured in Matrigel under appropriate hormonal stimulation express typical mammary alveolar epithelial markers and exhibit morphological signatures of alveolar-like differentiation.

### 3.2. Mature Adipocytes under Co-Culture Conditions Lose Their Adipogenic Features and Acquire an Epithelial Mammary Phenotype

**Figure 3:**
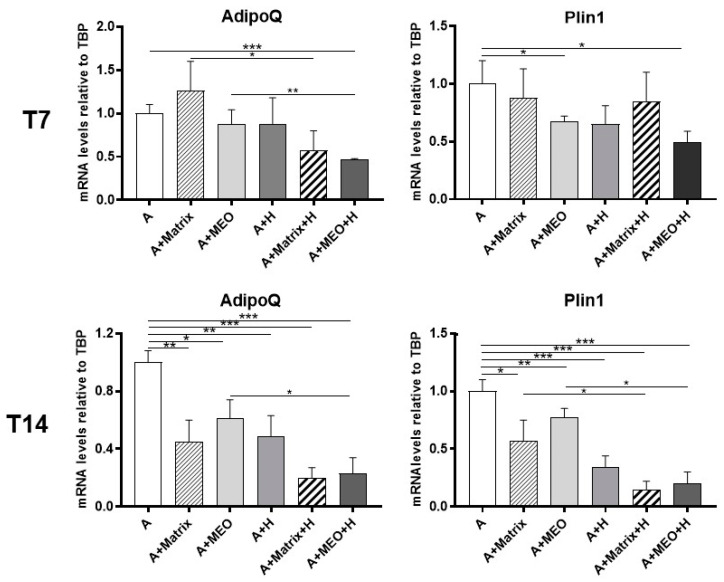
qRT-PCR analysis of adipogenic markers (AdipoQ and Plin1) in adipocytes co-cultured in absence/presence of T7 (top panel) and T14 (bottom panel) differentiated-MEO and in absence/presence of pregnancy hormones. Data (*n* = 3) are mean ± SEM; * *p* < 0.05, ** *p* < 0.01, *** *p* < 0.001 between biological groups as indicated. Data were analyzed using one-way ANOVA.

To assess whether hormone-treated MEOs can change the phenotype of differentiated adipocytes by releasing diffusible factors, we evaluated the gene expression of adipose markers in differentiated adipocytes co-cultured with hormone-treated MEOs. After 3 days of co-culture, Adiponectin (AdipoQ) and Perilipin 1 (Plin1) mRNA expression were significantly reduced in mature adipocytes co-cultured with T7-hormone-treated MEOs (A+MEO+H), highlighting that in this condition adipocytes lose some of their typical molecular signatures (Figure 3, upper panels). The reduction of the above adipose markers was even more evident after 14 days of co-culture with hormone-treated MEOs, when a significant reduction of these adipocyte markers was also detected in all the other conditions (A+Matrix, A+MEO, A+H, A+Matrix+H) (Figure 3, lower panels). This suggests that while MEO is crucial at T7, at T14 the presence of Matrigel alone also reduces the gene expression of adipocyte markers, possibly due to its content of growth factors, which are also produced by MEO (Figure S4).

**Figure 4:**
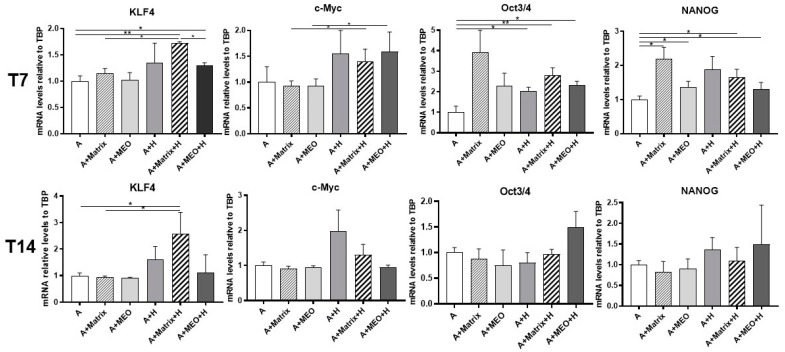
qRT-PCR analysis of reprogramming markers in adipocytes co-cultured in absence/presence of T7 (top panel) and T14 (bottom panel) differentiated-MEO and in absence/presence of pregnancy hormones. Data (*n* = 3) are mean ± SEM; * *p* < 0.05, ** *p* < 0.01 between biological groups as indicated. Data were analyzed using one-way ANOVA.

Previous data published by our group showed that adipo-epithelial transdifferentiation is accompanied by transient expression of genes typical of stem cells that are in turn involved in the reprogramming of mature cells into pluripotent cells [8]. Thus, to assess whether adipocytes reprogram their genome under our experimental conditions, we performed a gene expression analysis of the main markers of gene reprogramming, as KLF4, c-Myc, Oct3/4, and NANOG. Except c-Myc, all genes significantly increased in mature adipocytes co-cultured with T7-hormone-treated MEOs (A+MEO+H) and in the presence of Matrigel and/or hormones (A+Matrix, A+H, A+Matrix+H). In striking contrast, KLF4 was the only gene found to increase significantly at T14 specifically in the condition with Matrigel and hormones (A+Matrix+H) (Figure 4).

These data suggest that in our experimental setting adipocyte reprogramming is an early event, possibly preceding cellular transdifferentiation.

**Figure 5:**
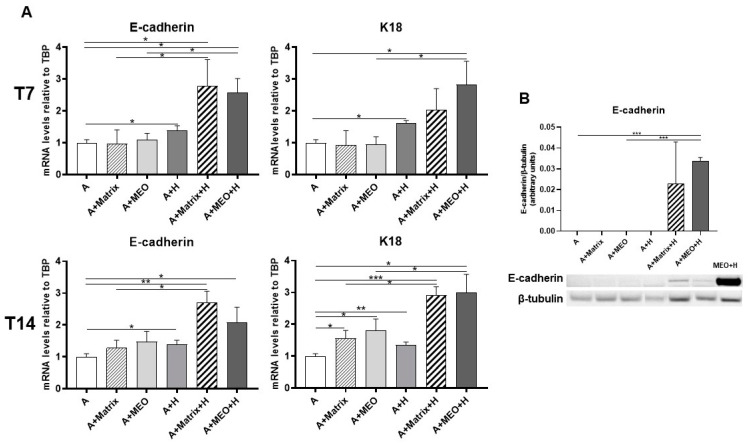
qRT-PCR analysis of epithelial markers (**A**) and representative immunoblot and quantification of E-cadherin expression (**B**) in adipocytes co-cultured in absence/presence of T7 (top panel) and T14 (bottom panel) differentiated-MEO and in absence/presence of pregnancy hormones. Data (*n* = 3) are mean ± SEM; * *p* < 0.05, ** *p* < 0.01, *** *p* < 0.001 between biological groups as indicated. Data were analyzed using one-way ANOVA. In B, MEOs treated with hormones for 14 days (MEO+H) were used as positive control.

To assess whether adipocytes co-cultured with MEOs and exposed to pregnancy hormones not only lose their adipose phenotype but also transdifferentiate toward an epithelial phenotype, gene expression analysis of typical epithelial markers was performed. After 3 days of co-culture, E-cadherin, and K18 increased significantly in adipocytes treated with T7-differentiated MEOs and hormones (A+MEO+H), compared to untreated adipocytes (Figure 5A, upper panels). Interestingly, E-cadherin increased also when the MEOs were absent (A+Matrix+H) or in the presence of only hormones (A+H), highlighting the importance of the hormonal stimulation and of growth factors present in Matrigel in inducing the adipo-epithelial transdifferentiation. Instead, K18 not only increased in the presence of MEOs and hormones (A+MEO+H) but also in the presence of hormones alone (A+H). It is noteworthy that MEOs alone were not able to induce the expression of epithelial markers at T7.

Similar data were obtained at T14, further confirming the involvement of MEOs, Matrigel, and hormones as factors contributing to the increase in gene expression of epithelial markers (Figure 5A, lower panels).

Western blotting experiments performed on adipocyte protein extracts confirmed the expression of E-cadherin protein in adipocytes co-cultured with MEOs or Matrigel and treated with hormones to a similar extent (Figure 5B).

**Figure 6:**
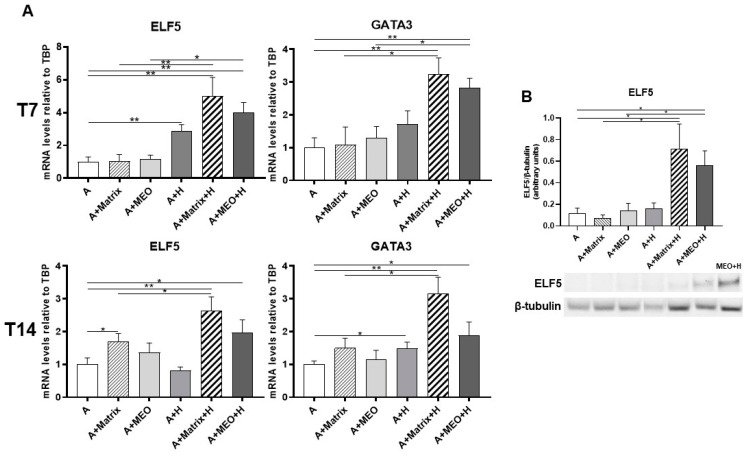
qRT-PCR (**A**) analysis of pinking markers and representative immunoblot and quantification of ELF5 expression (**B**) in adipocytes co-cultured in absence/presence of T7 (top panel) and T14 (bottom panel) differentiated-MEO and in absence/presence of pregnancy hormones. Data (*n* = 3) are mean ± SEM; * *p* < 0.05, ** *p* < 0.01 between biological groups as indicated. Data were analyzed using one-way ANOVA. In **B**, MEO treated with hormones for 14 days (MEO+H) were used as positive control.

Lastly, we assessed whether adipocytes co-cultured with hormones and MEOs express molecular signatures of mammary alveolar epithelial cells. We thus performed a gene expression analysis of typical markers of early lactogenesis, ELF5 and GATA3, and of late lactogenesis, β-casein and WAP. ELF5 and GATA3 mRNA significantly increased in adipocytes treated with hormones and in adipocytes co-cultured with T7 and T14-differentiated MEOs and hormones (Figure 6A). ELF5 and GATA3 gene expression also increased in all the other conditions (A+Matrix, A+H, A+Matrix+H).

Western blotting analysis confirmed the expression of ELF5 protein in adipocytes co-cultured with MEOs or Matrigel treated with hormones (A+MEO+H, A+Matrigel+H) (Figure 6B).

**Figure 7:**
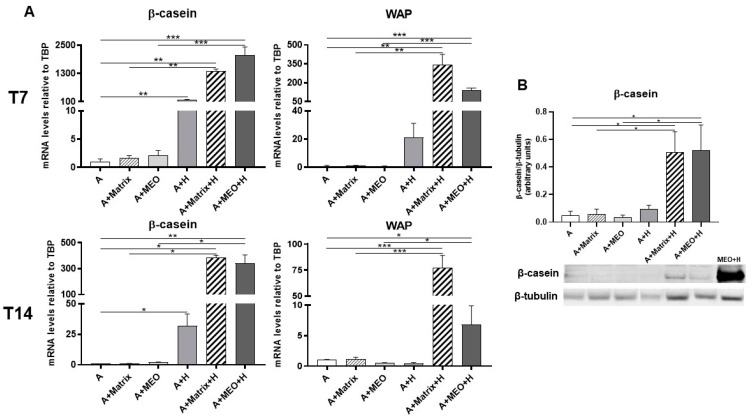
qRT-PCR (**A**) analysis of milk markers and representative immunoblot and quantification of β-casein expression (**B**) in adipocytes co-cultured in absence/presence of T7 (top panel) and T14 (bottom panel) differentiated-MEO and in absence/presence of pregnancy hormones. Data (*n* = 3) are mean ± SEM; * *p* < 0.05, ** *p* < 0.01, *** *p* < 0.001 between biological groups as indicated. Data were analyzed using one-way ANOVA. In (**B**), MEOs treated with hormones for 14 days (MEO+H) were used as positive control.

β-casein and WAP mRNA significantly increased in adipocytes treated with hormones and in adipocytes co-cultured with MEOs at T7 and T14 of differentiation plus and hormones (Figure 7A). The data were confirmed by protein expression analysis (Figure 7B). Interestingly, β-casein and WAP were also detectable when MEOs were absent, confirming the importance of both hormonal stimulation and Matrigel growth factors in inducing the production of milk proteins.

In conclusion, these results, show that our experimental setting is a suitable approach to reproduce mammary gland adipocyte-epithelial transdifferentiation in vitro, which appears to be driven by diffusible factors, including pregnancy hormones and factors released by MEOs and Matrigel. These diffusible factors produced by MEOs and/or Matrigel may act in concert with pregnancy hormones to drive adipocyte-epithelial transdifferentiation in our experimental setting.

### 3.3. MEOs and Mammary Adipocytes Release miRNAs in the Co-Culture Supernatant

**Figure 8:**
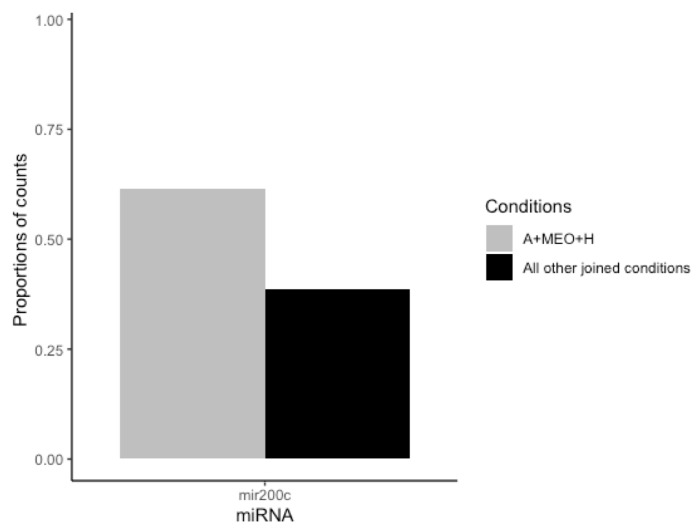
Bar plot of mir200c miRNA (*x*-axis) representing the proportions of counts specific to the considered conditions: A+MEO+H and All other joined conditions (A, A+H, A+MEO, A+Matrix, A+Matrix+H) (*y*-axis).

MiRNAs are diffusible factors playing pleiomorphic functions also during development [25]. Thus, to assess whether miRNAs are involved in the crosstalk between MEOs and adipocytes, possibly affecting adipocyte-epithelial transdifferentiation, we analyzed their presence in the supernatant. Differentially expressed miRNA were identified considering only entities with biotype as “miRNA”. The differential analysis was conducted considering: (1) transdifferentiating condition (A+MEO+H) vs. each single condition alone (A, A+H, A+MEO, A+Matrix, A+Matrix+H); (2) transdifferentiating condition (A+MEO+H) vs. all other joined conditions (A, A+H, A+MEO, A+Matrix, A+Matrix+H), and (3) transdifferentiating conditions (A+MEO+H and A+Matrix+H) vs. non-transdifferentiating ones (A, A+H, A+MEO, A+Matrix) since expression data showed that both A+MEO+H and A+Matrix+H induce the adipocyte-epithelial transdifferentiation. None of the emerged miRNAs was differentially expressed after correction for multiple testing. However, one candidate, mir200c, emerged from (A+MEO+H) vs. all other joined conditions (A, A+H, A+MEO, A+Matrix, A+Matrix+H) and showed a differential expression with a nominal statistical significance (non-adjusted *p*-value ≤ 0.05) supported by consistent sequencing data (more than 20 mapped reads in at least 3 samples) (Figure 8).

### 3.4. hMADS Adipocytes Acquire Adipo-Epithelial Transdifferentiation Markers under Pregnancy Stimuli

**Figure 9:**
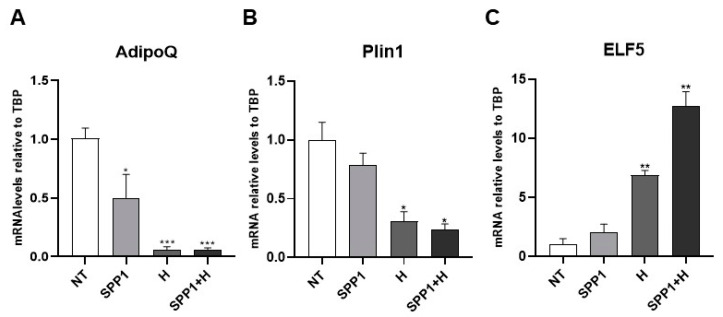
qRT-PCR analysis of AdipoQ (**A**), Plin1 (**B**) and ELF5 (**C**), in hMADS adipocytes maintained for 24 h under osteopontin 500 ng/mL (SPP1), pregnancy hormones (H) and H+SPP1. Data (*n* = 3) are mean ± SEM; * *p* < 0.05, ** *p* < 0.01, *** *p* < 0.001 compared with untreated hMADS adipocytes (NT). Data were analyzed using one-way ANOVA.

In a study aimed at identifying key adipo-epithelial transdifferentiation factors, we compared the gene expression profile of a cleared mammary fat pad to an intact mammary gland in virgin and pregnant mice [8]. Bioinformatic analysis pointed toward SPP1 as a potential candidate responsible for the adipo-epithelial conversion [8], consistent with data from the literature supporting its crucial role in alveologenesis [26,27]. Interestingly, targeted inhibition of osteopontin expression in the mammary gland causes abnormal morphogenesis and lactation deficiency [28]. Based on these premises, we differentiated human adipose-derived stem cells (hMADS) into mature adipocytes and treated them with SPP1, pregnancy hormones, or both for 24 h to detect molecular signatures possibly suggestive of adipo-epithelial transdifferentiation. While SPP1 treatment alone only significantly reduced AdipoQ mRNA levels, pregnancy hormones with or without SPP1 induced a significant reduction of both AdipoQ and Plin1 adipogenic markers. Importantly, in these conditions the adipose phenotype loss was paralleled by a significant increase in the alveolar glandular marker ELF5. Interestingly, while the hormone treatment was sufficient to reduce adipogenic marker expression, SPP1 appeared to exhibit a synergistic role with hormones in inducing the increased expression of the alveologenesis marker (Figure 9).

## 4. Discussion

It is well known that mature adipocytes of the subcutaneous depots are required for a normal development of the mammary gland during pregnancy and lactation [29]. Our previous data suggested a reversible transdifferentiation of adipocytes into mammary alveolar cells during pregnancy, lactation, and the post-lactation period in mice [8]. Interestingly, an independent laboratory has recently shown that a small percentage of myoepithelial cells of mammary gland alveoli derive from adipocytes by transdifferentiation [30], further supporting the occurrence of cellular transdifferentiation processes in the developing mammary gland [2,3]. However, a recent paper mainly based on lineage tracing data questioned such phenomenon [9], which will surely deserve further studies with sophisticated techniques and experimental approaches in the future.

Previous data showed that, although adipocytes express prolactin and progesterone receptors that coordinate the alveolar formation [31,32], the hormonal milieu of pregnancy in vivo is not sufficient per se to induce adipo-epithelial transdifferentiation. Indeed, cleared fat pads (i.e., fat of the breast remaining after ductal surgical removal) [33] of pregnant mice did not show signs of alveologenesis despite a normal development of the contralateral mammary glands [8]. This suggests that one or more paracrine factors should be secreted by ductal cells and our microarray study showed that osteopontin could play a key role in this phenomenon [8]. Of note, targeted inhibition of SPP1 expression in the mammary gland causes abnormal morphogenesis and lactation deficiency [28], whereas its targeted overexpression drives alveologenesis [26]. Furthermore, SPP1 expression is regulated by its inhibitor RunX2, which is physiologically reduced in late pregnancy, and transgenic mice in which RunX2 is under the control of an MMTV promoter (MMTV-RunX2 mouse) show failure of lactation [34]. Interestingly, it has been shown that SPP1 plays a role in cell adhesion [35], a crucial step if adipocytes were to convert into alveolar cells.

A second gene that was among the top ten of those expressed in the above-mentioned microarray study was GATA3. This transcription factor is highly expressed in the ductal luminal cells as well as in the terminal buds of the mammary gland and plays an important role in the development and maintenance of luminal epithelial cells in the mammary gland [36]. Adipocyte differentiation is suppressed by forced expression of GATA3 [37]. Furthermore, GATA3 and other GATA family proteins are known as pioneering factors able to bind condensed chromatin and thereby to determine cell fate [38].

We also found that all ductal luminal epithelial cells, pink adipocytes, and some intermediate forms (adipocytes in early steps of conversion) showed a strong nuclear ELF5 staining by immunohistochemistry [8]. Importantly, (i) it has been demonstrated that ELF5 regulates the fate of luminal progenitor cells during pregnancy [39], which suggests that it may also play a role in the direct conversion of mammary adipocytes toward an epithelial phenotype; (ii) ELF5 possesses a pointed domain that is specialized for protein-protein interaction [40], suggesting that it may bind and recruit chromatin re-modelers to specific genomic loci that require activation for transdifferentiation; and (iii) even in virgin mice, forced ELF5 expression in mammary glands induces alveologenesis and the production of milk protein [38].

In conclusion, the emerging picture suggests that combined action of SPP1, ELF5, and GATA3 could be very important for adipo-epithelial conversion.

Data from the present study not only show that, under appropriate co-culture conditions with MEOs and Matrigel, it is possible to induce the adipo-epithelial conversion of mature adipocytes in vitro by mimicking the pregnancy milieu, but also that these converted adipocytes express very high levels of milk protein genes. Of note, the expression of reprogramming genes was also detected in these experimental conditions, further supporting the direct conversion of the cellular phenotype [41]. Interestingly, our data are in line with a previous study performed on goat preadipocytes showing that conditioned medium made from goat mammary epithelial cells (MECs) cultured on Matrigel induces transdifferention signatures of goat preadipocytes into MECs [42].

However, our results show that transdifferentiation in adipocytes also occurs to some extent when adipocytes are treated with hormones and/or cultured with Matrigel even in the absence of MEOs. This suggests that growth factors present in Matrigel, including FGF, EGF, TGFβ, and IGF, play key roles in inducing adipo-epithelial transdifferentiation.

In this context, previous research has demonstrated similar outcomes when adipose-derived stem cells (ASCs) were cultured in a fibrin scaffold treated with EGF. This treatment led to the differentiation of ASCs into an epithelial phenotype expressing E-cadherin [43]. Furthermore, the presence of growth factors, including FGF, in Matrigel likely contributes to the induction of adipo-epithelial transdifferentiation. FGF signaling is known to play a pivotal role in mammary gland development and stem cell dynamics. For instance, deletion of FGFR2, expressed in basal and luminal MECs, results in defects in branching morphogenesis [44]. Additionally, FGF7 and FGF10 are crucial for ELF5 expression through the PI3-kinase/Akt pathway, further emphasizing the importance of FGF signaling in mammary gland development [45]. Moreover, IGF is implicated in various stages of mammary gland development. During puberty, IGF is associated with ductal elongation, while during lactation, it plays a role in cell survival and involution [46]. This underscores the multifaceted role of growth factors in regulating mammary gland development and function.

Our experimental setting represents a novel and suitable approach to reproduce in vitro mammary gland adipocyte-epithelial transdifferentiation, which appears to be driven by diffusible factors, including pregnancy hormones and factors released by MEOs and/or Matrigel. Likely, these diffusible factors act in concert with pregnancy hormones to drive adipocyte-epithelial transdifferentiation. Collectively, these findings emphasize the intricate interplay between the extracellular matrix components and diffusible factors in orchestrating the adipo-epithelial transdifferentiation process, shedding light on the multifaceted mechanisms underlying mammary gland development.

Based on our data, miRNAs could play a role in the regulation of this phenomenon. Mir200c emerged from the comparison of transdifferentiating (A+MEO+H) vs. all other joined conditions (A, A+H, A+MEO, A+Matrix, A+Matrix+H) and has been previously described as involved in epithelial-mesenchymal transition (EMT) [47]. In fact, enforced constitutive expression of mir200 in mesenchymal cells promoted EMT [10], and its loss correlates with a lack of E-cadherin expression in invasive breast cancer [11]. The Mir200 family plays a critical role in EMT targeting and in repressing the expression of ZEB1 and ZEB2, inhibitors of E-cadherin transcription [11]. Furthermore, mir-200 represses TGFβ enforcing the epithelial phenotype. TGFβ signaling, has been shown to play an important role in EMT [48]. In fact, adding TGFβ to epithelial cells in culture is a convenient way to induce EMT in various epithelial cells [49]. Lastly, these data are in line with the high levels of mir200 in late stages of pregnancy in mice [50].

In conclusion, our data provide an in vitro model to study adipo-epithelial transdifferentiation and stress the role of SPP1, ELF5, GATA3, and mir200c as key candidates regulating this phenomenon.

## Figures and Tables

**Table 1:** Experimental conditions.

Experimental Condition	Acronym
Adipocytes	A
Adipocytes and hormones	A+H
Adipocytes and MEOs (embedded in Matrigel)	A+MEO
Adipocytes and MEOs (embedded in Matrigel) and hormones	A+MEO+H
Adipocytes and Matrigel (without MEOs)	A+Matrix
Adipocytes and Matrigel (without MEOs) and hormones	A+Matrix+H

**Table 2:** Taqman probes all from Applied Biosystems #4453320.

Target Gene	Assay ID
AdipoQ	Mm00456425_m1
AdipoQ	Hs00977214_m1
FGF	Mm01285715_m1
β-casein	Mm00839913_m1
c-Myc	Mm01192721_m1
E-cadherin	Mm01247357_m1
ELF5	Mm00468732_m1
ELF5	Hs00154971_m1
GATA3	Mm00484683_m1
IGF	Mm00439564_m1
K18	Mm01601704_g1
KLF4	Mm00516104_m1
NANOG	Mm02384862_g1
Oct3/4	Mm00305917_g1
Plin1	Mm00558672_m1
Plin1	Hs00160173_m1
RANK-L	Mm00441906_m1
SPP1	Mm00436767_m1
TBP	Mm00446973_m1
TGFβ	Mm01178820_m1
WAP	Mm00839664_m1
Wnt6	Mm00437353_m1

**Table 3:** Primary antibodies.

Antibodies	Host *	Diluition	Source
E-cadherin	R	1:200	Santa Cruz Biotechnology/sc-7870
ELF5	R	1:200	Thermo Fisher/720380
β-casein	R	1:100	Thermo Fisher/PA5-109599
β-Tubulin	M	1:800	Santa Cruz Biotechnology/sc-5274

* M, mouse; R, rabbit.

**Table 4:** Secondary antibodies.

Conjugated to	React *	Dilution	Source	ID
Peroxidase	M	1:5000	Jackson ImmunoResearch	715-036-150
Peroxidase	R	1:1000	Vector Laboratories	PI-1000

* M, mouse; R, rabbit.

## Data Availability

The data that support the findings of this study are available from the corresponding author upon reasonable request.

## Supplementary Materials

The following are available online at https://www.mdpi.com/article/10.3390/cells13110943/s1, Figure S1: Model of primary adipocytes and mammary epithelial organoids (MEO) co-culture, Figure S2: Schematic representation of MEO and primary preadipocytes, Figure S3: qRT-PCR analysis of Wnt6 and RANK-L expression, Figure S4: qRT-PCR analysis of FGF, TGFβ and IGF expression.
