# Supplementary material for: Adipo-Epithelial Transdifferentiation in In Vitro Models of the Mammary Gland
Source: Cells. 2024 May 30;13(11):943. doi: 10.3390/cells13110943 (PMC11171678; doi:10.3390/cells13110943)
Supplement: Supplementary file 1 [file cells-13-00943-s001.zip › cells-3036625-supplementary.pdf]

Supplementary figures

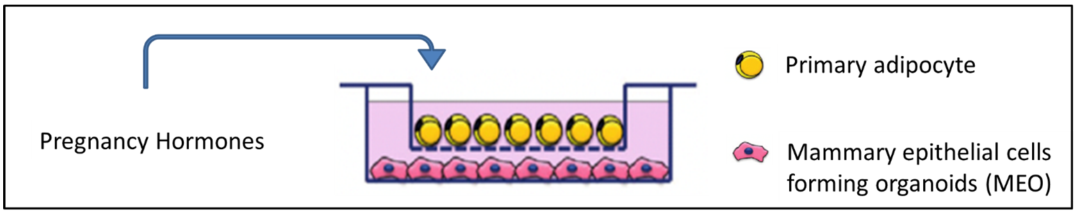

Figure S1: Model of primary adipocytes and mammary epithelial organoids (MEO) co-culture. MEO incorporated into Matrigel were cultured in the lower well and mature adipocytes in the upper insert. The adipocytes were cultivated within the transwell with a 0.4 μm membrane that allows the exchange of culture medium and diffusible factors, not allowing cell migration between the two compartments. MEO and adipocytes were co-cultured for 3 days in the presence/absence of the pregnancy hormones.

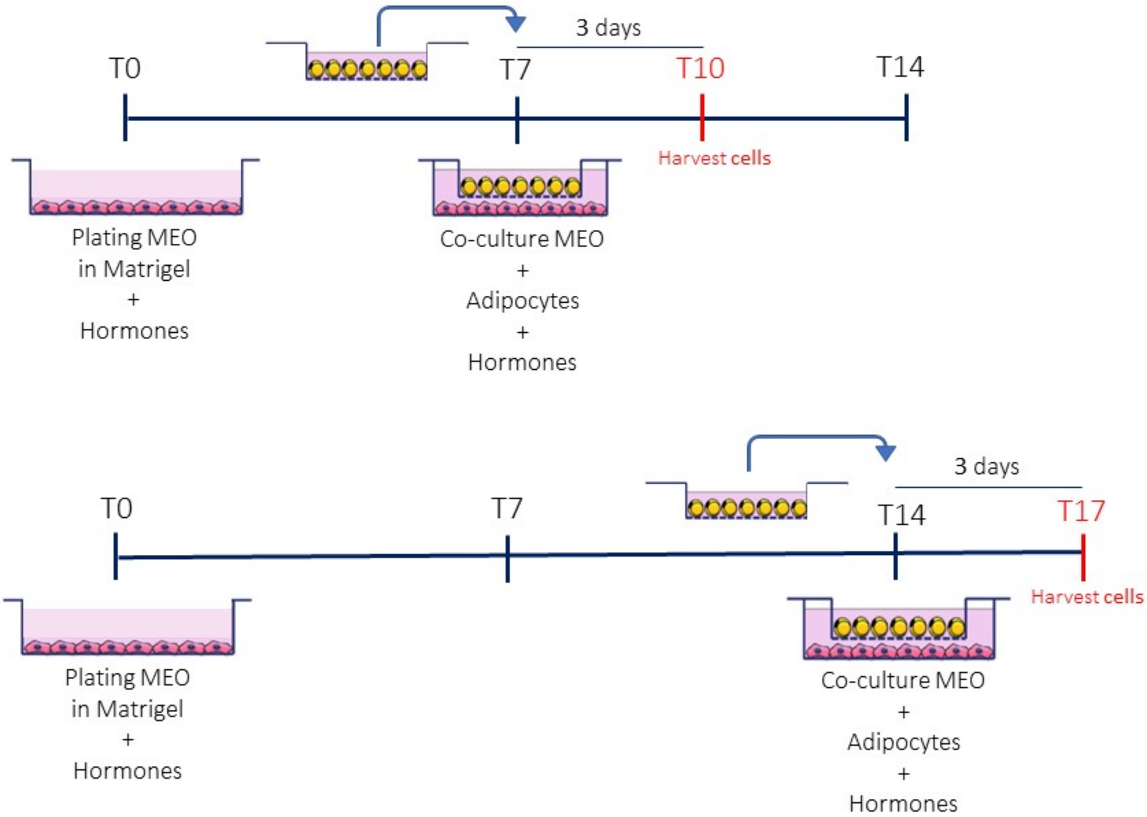

Figure S2: Schematic timeline representation of MEO and primary adipocytes seeding respectively on the basolateral and apical sides of the transwell to attain a co-culture *in vitro* model mimicking the microenvironment of the *in vivo* mammary gland.

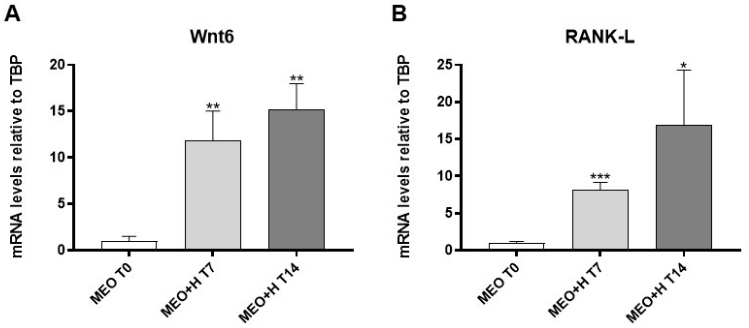

Figure S3: qRT-PCR analysis of Wnt6 (A) and RANK-L (B) expression in freshly isolated MEO (T0) and MEO at 7 and 14 days of culture under hormonal stimulation. Data (n=3) are mean ± SEM; * P<0.05, **P<0.01, ***P<0.001 compared with MEO T0. Data were analyzed using one-way ANOVA.

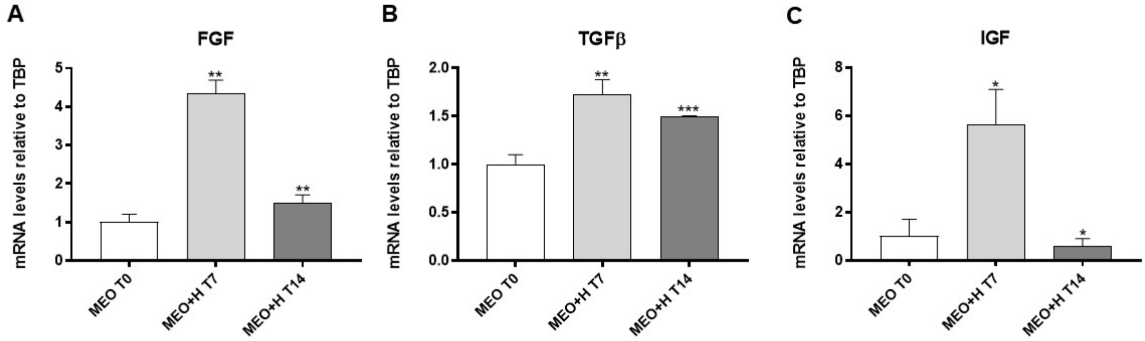

Figure S4: qRT-PCR analysis of FGF (A), TGFβ (B) and IGF (C) expression in freshly isolated MEO (T0) and MEO at 7 and 14 days of culture under hormonal stimulation. Data (n=3) are mean ± SEM; * P<0.05, **P<0.01, ***P<0.001 compared with MEO T0. Data were analyzed using one-way ANOVA.
